# Predictors for prolonged and escalated perioperative antibiotic therapy after microvascular head and neck reconstruction: a comprehensive analysis of 446 cases

**DOI:** 10.1186/s13005-024-00463-9

**Published:** 2024-10-15

**Authors:** Johannes G. Schuderer, Florian Hoferer, Jonas Eichberger, Mathias Fiedler, André Gessner, Florian Hitzenbichler, Maximilian Gottsauner, Michael Maurer, Johannes K. Meier, Torsten E. Reichert, Tobias Ettl

**Affiliations:** 1 Department of Oral and Maxillofacial Surgery, University Hospital Regensburg, Franz-Josef-Strauß-Allee 11, Regensburg, 93053 Germany; 2 Institute of Clinical Microbiology and Hygiene, University Hospital Regensburg, Regensburg, Germany; 3 Department of Infection Prevention and Infectious Diseases, University Hospital Regensburg, Regensburg, Germany

**Keywords:** Head neck surgery, Microvascular reconstruction, Perioperative antibiotic prophylaxis, Surgical site infections

## Abstract

Literature suggests that intravenous prophylaxis exceeding 48 h offers no additional benefit in preventing surgical site infections (SSI) in patients with microvascular head and neck reconstruction. However, protocols for antibiotic therapy duration post-reconstruction are not standardized. This study identifies factors predicting prolonged intravenous antibiotic use and antibiotic escalation in patients receiving free flap head neck reconstruction.

A retrospective analysis of 446 patients receiving free flap reconstruction was conducted, examining predictors for antibiotic therapy > 10 days and postoperative escalation. 111 patients (24.8%) experienced escalation, while 159 patients (35.6%) received prolonged therapy. Multivariate regression analysis revealed predictors for escalation: microvascular bone reconstruction (*p* = 0.008, OR = 2.0), clinically suspected SSI (*p* < 0.001, OR = 5.4), culture-positive SSI (*p* = 0.03, OR = 2.9), extended ICU stay (*p* = 0.01, OR = 1.1) and hospital-acquired pneumonia (*p* = 0.01, OR = 5.9). Prolonged therapy was associated with bone reconstruction (*p* = 0.06, OR = 2.0), preoperative irradiation (*p* = 0.001, OR = 1.9) and culture-positive SSI (*p* < 0.001, OR = 3.5).

The study concludes that SSIs are a primary factor driving the escalation of perioperative antibiotic use. Clinical suspicion of infection often necessitates escalation, even in the absence of confirmed microbiological evidence. Microvascular bone reconstruction was a significant predictor for both the escalation and extension of antibiotic therapy beyond 10 days. Furthermore, preoperative radiation therapy, hospital-acquired pneumonia, and prolonged ICU stay were associated with an increased likelihood of escalation, resulting in significantly extended antibiotic administration during hospitalization. Antibiotic stewardship programmes must be implemented to reduce postoperative antibiotic administration time.

**Trial registration **The study was registered approved by the local Ethics Committee (Nr: 18-1131-104).

## Background

In recent years, the success rates of microvascular transplants in the reconstruction of head and neck defects have consistently surpassed the ninety percent mark [1–3]. This achievement can be attributed to the increasing experience in specialized centres of head and neck cancer and reconstructive surgery, and to the increasing supply of educational programs, courses and fellowships.

However, the occurrence of post-operative surgical site infections (SSI), a complication known to adversely affect the outcome of reconstructive interventions, has remained a focal point of concern [4, 5]. Most reconstructive head neck procedures are classified as clean-contaminated, involving the dissection of mucosa or opening the respiratory tract [6]. Previously treated patients with tumor recurrence or infections of the jaw carry changes in their oral microbiome and are prone to infections with gram negative inherently penicillin resistant bacteria [4, 7–12]. This must be taken into account when selecting an appropriate perioperative antibiotic regimen anticipating the manifestation of surgical site infections after microvascular transplantation.

Numerous studies have investigated the advantages of perioperative prophylactic antibiotics [4, 5]. However, recent findings increasingly indicate that antibiotic treatment itself may influence the success of adjuvant radio or checkpoint therapy treatment and moderate survival outcomes in tumor patients [13, 14]. Additionally, it is widely known that antibiotic administration can lead to side effects, such as Clostridioides difficile infection and antibiotic-associated diarrhoea [15]. Notably, several prominent studies have demonstrated that intravenous perioperative prophylaxis exceeding 48 h does not hold benefits avoiding SSI while patient groups presenting risk factors have been usually prescribed a more liberal antibiotic regimen [16, 17]. Nevertheless, there are currently no standardized protocols specifying the duration after reconstruction in this patient group.

The aim of this study was to identify factors predicting prolonged intravenous perioperative antibiotic administration and predictors for antibiotic therapy escalation in patients with free flap reconstruction in the head and neck area.

## Material and methods

**Fig. 1:**
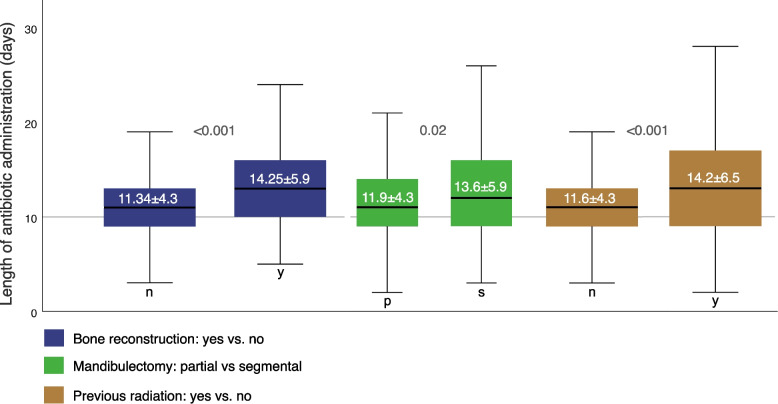
STROBE flowchart (Strengthening the Reporting of Observational Studies in Epidemiology)

The medical records of patients who underwent microvascular flap reconstruction after clean and clean- contaminated head neck surgery between 2016 and 2024 were retrospectively examined [18]. Patients with incomplete documentation or a complex postoperative course requiring transfer to another department were excluded. All surgical patients were admitted through our department, operated on, and discharged from our ward after the appropriate length of stay. With regard to the report of observational research, a STROBE (Strengthening the Reporting of Observational Studies in Epidemiology) [19] flowchart was created to outline the selection, inclusion and analysis of the study participants (Fig. 1).

The duration of perioperative antibiotic administration was defined from the initial intraoperative administration (documented on the aesthetic protocol) until postoperative administration was ceased or an antibiotic escalation occurred.

Regarding inpatient antibiotic administration, an analysis was conducted on the specific antibiotic agents used, duration of intravenous administration and escalation. A priori, due to surgical standard operation procedures (SOPs), which are designed to standardize perioperative antibiotic administration intravenous administration of amoxicillin/clavulanic acid or ampicillin/sulbactam was conducted as the standard procedure. In cases of allergy, antibiotics from the cephalosporin or lincosamide classes were employed. Directly associated side effects of prolonged antibiotic administration, such as acute kidney failure, Clostridioides difficile infection and antibiotic-associated diarrhoea or allergic reactions were not part of the data analysis.

Due to missing guidelines regarding the exact duration of perioperative antibiotic coverage in free flap reconstruction in our department, the prescribed antibiotic duration varied widely. The transition between perioperative prophylaxis and therapy remains undefined in terms of duration. In our department, patients with a history of radiation therapy, microvascular bone reconstruction or extensive soft tissue resection received prolonged postoperative antibiotic coverage to provide adequate infectious protection up to 10 days. To identify predictors that might justify antibiotic administration exceeding 10 days without escalation in microvascular reconstructions, a 10-day baseline was statistically established. Antibiotic escalation was defined as a shift from perioperative antibiotic therapy to either a higher-class, more broad-spectrum antibiotic or a targeted adjustment based on an antibiogram [20].

SSIs were defined following Centers for Disease Control and Prevention (CDC) criteria [21]. If a clinical SSI was assumed swaps with microbiological diagnostics and antibiotic susceptibility testing according to European Committee on Antimicrobial Susceptibility Testing (EUCAST)[20] guidelines was performed. If no microbiological growth was detected, antibiotic treatment was escalated based on clinical parameters [22], and the SSI was classified as culture-negative. Remote infections such as pneumonia were documented based on discharge letter coding, only considering infections during hospital stay. Therefore, three different SSI variables were made: clinical suspected SSI, culture positive SSI and culture negative SSI.

Various epidemiological factors, surgical and anaesthesiologic parameters were assessed, including age, sex, American Society of Anaesthesiologists (ASA) score, operative duration, duration of intensive care unit (ICU) and regular ward stay (NW), defect localization, flap type, mandibular and maxillary resections, neck dissection, tracheotomy, preoperative radiotherapy, transfusion and haemoglobin values preoperatively, first, second and third day after surgery (mg/dl). Flap success and flap loss, with local flap complications, such as venous or arterial congestion with need for operative revision were documented. Flap loss was defined as complete or partial necrosis of the flap with consequent impaired local reconstruction.

We conducted univariate analyses utilizing Fisher’s exact, Chi-square and Student’s T-test, scale level adjusted. Logistic (binary) regression analysis was employed to identify predictor variables, using the maximum likelihood method for regression coefficients (B) and Odds ratios (OR). Multiple regression models were built, employing analysis of variance (ANOVA) for hypothesis testing. We calculated regression coefficients (B) and determined significance levels of the F-test and effect sizes (f2) considering f2 ≥ 0.35 as indicative of a strong effect. Additionally, we computed 95% confidence intervals (CI) and considered a significance level of P ≤ 0.05 as statistically significant. All analyses were performed using SPSS version 29.0 (IBM Corp.). Clinically relevant parameters identified in univariate analysis were utilized in the model.

The primary statistical endpoints were defined as antibiotic escalation and antibiotic therapy without antibiotic escalation lasting longer than 10 days. Total length of antibiotic therapy was set as secondary endpoint. Binary logistic and linear regression analyses were conducted, incorporating these endpoints as dependent variables.

## Results

The study included 481 patients who underwent free flap reconstruction in the head and neck region between 2016 and 2024. Due to incomplete data documentation or dropout variables, a total of 446 patients were included in the retrospective analysis. For epidemiological and surgical parameters see Table 1. In total, six double flaps were performed, as detailed in Table 1, with combinations of two flaps each. These included four instances of Fibula + Anterolateral Thigh (ALT) and two instances of Fibula + Radial Forearm.

**Table 1:** Epidemiologic characteristics and univariate analysis

		Overall *N* = 446	Escalation *N* = 111	*P* value	AB > 10 d *N* = 159	*P* value
Sex				0.1		0.6
	male	285 (63.9%)	65 (58.6%)		102 (64.2%)	
	female	161 (36.1%)	46 (34.3%)		57 (35.8%)	
Age	years Ø	63.4 ± 11.4	64.3 ± 12.6		63.3 ± 11.2	0.2
Diagnosis				**0.001**		0.2
	OSCC	362 (81.2%)	77 (69.4%)		135 (84.9%)	
	IORN	41 (9.2%)	21 (18.9%)		10 (6.3%)	
	MRONJ	18 (4.0%)	5 (4.5%)		8 (5%)	
	Osteomyelitis	16 (3.6%)	4 (3.6%)		6 (3.8%)	
	others	9 (2.0%)	4 (3.6%)		-	
Flap type				** < 0.001**		**0.02**
	RFF	195 (43.7%)	36 (32.4%)		65 (40.9%)	
	FFF	130 (29.1%)	45 (40.5%)		48 (30.2%)	
	ALT	77 (17.3%)	12 (10.8%)		30 (18.9%)	
	LAT	17(3.8%)	5 (4.5%)		4 (2.4%)	
	Scapula	12 (2.7%)	4 (3.6%)		7 (4.5%)	
	Double flap	6 (1.3%)	6 (5.4%)		-	
	UA	5 (1.1%)	-		4 (2.5%)	
	DCIA	4 (0.9%)	3 (2.7%)		1 (0.6%)	
Wound Classification	clean-contaminated	408 (91.5%)	108 (97.3%)	**0.01**	152 (95.6%)	**0.001**
Neck Dissection	yes	310 (69.5%)	65 (58.6%)	** < 0.01**	117 (73.6%)	0.9
Bone resection				** < 0.01**		0.4
	segmental	143 (32.1%)	48 (43.2%)		50 (31.4%)	
	partial	92 (20.6%)	23 (20.7%)		34 (21.4%)	
Bone reconstruction	yes	152 (34.1%)	62 (55.9%)	** < 0.001**	56 (35.2%)	** < 0.01**
Tracheotomy	yes	219 (49.1%)	62 (55.9%)	0.1	85 (53.5%)	**0.02**
Skin size	cm^2^	53.5 ± 49.4	61.6 ± 54.5	**0.05**	51.9 ± 34.4	0.6
Flap success	yes	432 (96.9%)	103 (92.8%)	**0.05**	155 (97.5%)	0.3
Operation time	min Ø	385.5 ± 111.2	391.5 ± 97.9	0.5	396.8 ± 114.6	**0.05**
ICU	days Ø	2.8 ± 2.9	4.0 ± 4.4	** < 0.001**	2.65 ± 2.1	0.1
NW	days Ø	12.8 ± 5.9	15.5 ± 7.7	** < 0.001**	13.3 ± 5.4	** < 0.001**
LOS total	days Ø	15.7 ± 6.6	19.5 ± 8.1	** < 0.001**	15.9 ± 5.6	** < 0.001**
Radiation	yes	127 (28.5%)	49 (44.1%)	** < 0.001**	41 (25.8%)	0.2
Transfusion	yes	112 (25.1%)	41 (36.9%)	**0.001**	40 (25.2%)	-
Clinical SSI	yes	136 (30.5%)	68 (61.3%)	** < 0.001**	38 (23.9%)	0.2
Culture positive SSI	yes	109 (24.4%)	61(55%)	** < 0.001**	25(15.7%)	0.5
Culture negative SSI	yes	27 (6%)	7 (6%)	**0.005**	13 (8.2%)	0.3
HAP	yes	15 (3.4%)	11 (10%)	** < 0.001**	1 (0.6%)	0.4
ASA	mode	3				**0.02**
	≤ 2	193 (43.3%)	35 (31.5%)	0.1	68 (42.8%)	
	≥ 3	253 (56.7%)	76 (68.5%)	**0.04**	91 (57.2%)	
HB preoperative	mg/dl Ø	13.2 ± 2.0	12.8 ± 2.2	**0.02**	13.4 ± 1.9	0.9
HB 1. Day postoperative	mg/dl Ø	10 ± 1.6	9.6 ± 1.5	**0.01**	10.1 ± 1.6	0.6
HB 2. day postoperative	mg/dl Ø	9.4 ± 1.6	8.9 ± 1.4	** < 0.01**	9.4 ± 1.4	0.09
HB 3. day postoperative	mg/dl Ø	9.5 ± 1.6	8.9 ± 1.4	**0.001**	9.5 ± 1.5	0.1
Calculated perioperative Antibiotic				0.5		0.3
	Aminopenicillin	407 (91.3%)	98 (88.2%)		140 (89.2%)	
	Cephalosporine	15 (3.4%)	4 (3.6%)		6 (3.8%)	
	Piperacillin/Tazobactam	15 (3.3%)	6 (5.4%)		7 (4.5%)	
	Clindamycin	7 (1.6%)	3 (2.8%)		2 (1.3%)	
	Meropenem	2 (0.4%)	-		2 (1.3%)	
Escalated Antibiotic						
	Piperacillin/Tazobactam		59 (53.1%)		-	
	Ciprofloxacin		21 (18.9%)		-	
	Meropenem		10 (9%)		-	
	others		13 (11.7%)		-	
Total length of antibiotic duration	days Ø	12.3 ± 5.1	17.3 ± 6.1		13.2 ± 2.8	
Point of antibiotic escalation	days Ø		7.5 ± 4.6			
Antibiotic Oralization	yes	37 (8.3%)	3 (2.7%)		16 (10.1%)	

*ICU* Intensive care unit, *NW* Normal ward, *LOS* Length of stay, *SSI* Surgical site infection, *HAP* Hospital acquired pneumonia, *ASA* Physical status classification system of American Society of Anaesthesiologists, *HB* Haemoglobin, *RFF* Radial forearm flap, *FFF* Free fibula flap, *ALT* Anterior lateral thigh flap, *LAT* Latissimus flap, *UA* Upper arm flap, *DCIA* Deep circumflex iliac artery bone flap

Evaluation of perioperative antibiotics, showed that aminopenicillins (91.3%), cephalosporins (3.4%), piperacillin/tazobactam (3.3%), clindamycin (1.6%), and meropenem (0.4%) were administered. Only 8.3% of patients received oral antibiotic administration averaging on day 8.9 ± 3.9. The median duration of perioperative antibiotic therapy in the retrospective analysis was 10 days. The total duration of antibiotic therapy was 12.3 ± 5.1 days on average. A total of 408 (91.5%) patients underwent clean-contaminated surgery, of which 159 (35.7%) received perioperative antibiotics for longer than 10 days averaging 13.2 ± 2.8 days of antibiotic application. A total of 38 patients (23.9%) from the group receiving antibiotic therapy for more than 10 days developed a clinically confirmed SSI, and one patient (0.6%) developed pneumonia. Overall, 121 patients (27.1%) received postoperative antibiotic therapy for longer than 10 days without any documented indication for the prolonged duration. Postoperative antibiotic escalation was required in 111 (24.9%) patients, with an average escalation time of 7.5 ± 4.5 days. Total antibiotic duration in patients with antibiotic escalation was 17.3 ± 6.1 days.

A total of 136 (30.5%) patients were swapped due to clinical suspected surgical site infection (SSI) resulting in 109 (80.1%) cultural positive and 27 (6%) cultural negative SSIs. Exactly 50% (*n* = 68) of the patients with clinical assumed SSI were escalated. In the group of culture-positive SSIs (*n* = 109), 61 (55.9%) patients were escalated and 48 (44.1%) were not. In 27 (6%) cases, there was a culture-negative SSI, which was escalated 7 (6%) times. In the group without a swab, which means without clinically suspected infection, 43 (13.9%) patients were escalated for other reasons than SSI. Of these cases, 46% involved resection located in the lower jaw, 37 (86%) were tumor patients and MRONJ 4 (9.3%) reconstructed with radial forearm (39.5%) and fibula flaps (32.6%). 6 (14%) patients from this group developed hospital-acquired pneumonia. Additionally, 50 patients without a culture-positive SSI were escalated (difference from culture-negative SSIs = 7) (see Table 1). Regarding antibiotic escalation in this group, 6 patients from the flap complication group underwent antibiotic escalation due to flap complications, despite the absence of clinical signs of a surgical site infection.

Next to a total of 14 (3.1%) flap losses, 48 (10.8%) postoperative complications of the microvascular flap, with need for operative revision were documented. Venous congestion was observed in 25 (52%) cases, arterial issues in 11 (23%) cases, and in 12 (25%) cases, no specific cause was documented.

Among the patients who required antibiotic escalation (*N* = 111), piperacillin/tazobactam (53.1%), ciprofloxacin (18.9%), meropenem (9%), and others (11.7%) were used as preparations. Among the culture-positive SSIs, the following pathogens were identified: Enterobacter cloacae (20.5%), Staphylococcus spp. (11.1%), Candida spp. (10.3%), Streptococcus spp. (6.8%), Klebsiella spp. (6.8%), Citrobacter spp. (4.3%), Escherichia coli (4.3%), Pseudomonas spp. (3.4%), Serratia spp. (2.6%), Proteus spp. (1.7%), Corynebacteria (1.7%), Enterococci and Actinomyces (0.9%), and anaerobes (1.7%). In 19.8% of cases, no microbial growth was detected, leaving the clinically diagnosed SSI culture-negative.

### Univariate analysis

**Fig. 2:**
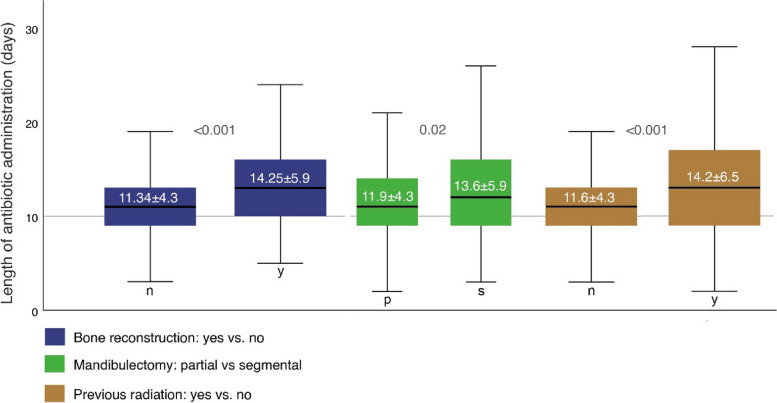
Boxplot analysis showing correlation of antibiotic therapy duration with bone reconstruction, mandibulectomy and previous radiation with *p* values

Regarding the primary endpoint of postoperative antibiotic escalation, the following factors were found to be statistically significant: Diagnosis (*p* = 0.001), flap type (*p* < 0.001), neck dissection (*p* < 0.01), bone resection (< 0.01), bone reconstruction (*p* < 0.001), skin size (*p* = 0.05), flap success (*p* = 0.05), duration of stay in the ICU (*p* < 0.001) and normal ward (*p* < 0.001), total length of hospital stay (*p* < 0.001), preoperative radiation (*p* < 0.001), transfusion (*p* = 0.001), surgical site infection (*p* < 0.001), hospital-acquired pneumonia (*p* < 0.001), ASA score ≥ 3 (*p* = 0.04) and haemoglobin levels (HB preoperative *p* = 0.02, HB first day postoperative *p* = 0.01, HB second day postoperative *p* < 0.01, HB third day postoperative *p* = 0.001). Regarding the second primary endpoint prolonged postoperative antibiotic duration > 10 days, the following factors were found to be statistically significant: flap type (*p* = 0.02), bone reconstruction (*p* < 0.01), tracheotomy (*p* = 0.02), operation time (*p* = 0.05), duration of stay on normal ward (*p* < 0.001) and total length of hospital stay (*p* < 0.001). Univariate analysis of total duration of antibiotic administration in days, a statistically significant correlation was found with done bone reconstruction (14.25 ± 5.9 vs. 11.34 ± 4.3, *p* < 0.001), segmental mandibulectomy (13.6 ± 5.9 vs. 11.9 ± 4.3, *p* = 0.02), perioperative radiation (14.2 ± 6.5 vs. 11.6 ± 4.3, *p* < 0.001) and the presence of a surgical site infection (15.7 ± 7.9 vs. 11.8 ± 4.3, *p* < 0.001) (see Table 1). To visualize the results boxplots were computed showing the length of antibiotic administration in days correlated to bone reconstruction, mandibulectomy and pre operative radiation displaying significances (Fig. 2).

### Multivariate analysis

Multivariate analyses to identify predictors of postoperative antibiotic escalation revealed that bone reconstruction (*p* = 0.008, OR = 2.011), clinical SSI (*p* < 0.001, OR = 5.469), cultural positive SSI (*p* = 0.03, OR = 2.9), length of stay in the ICU (*p* = 0.01, OR 1.121), and the presence of hospital-acquired pneumonia (*p* = 0.01, OR 5.925) were statistically significant. Radiation lost significance in this model (*p* = 0.2, OR = 1.424). Regression analysis regrading antibiotic therapy longer 10 days revealed bone reconstruction (*p* = 0.06, OR = 2.016) to be statistically relevant (Table 2). Multivariate linear regression analysis regarding total length of antibiotic therapy releveled previous radiotherapy (*p* < 0.001, B = 1.95), bone reconstruction (*p* < 0.001, B = 2,506) and SSI (*p* < 0.001, B = 3.586) to be statistically significant (Table 3).

**Table 2:** Multivariate binary logistic regression analysis

	Variable	Coding	***p***-value	OR	B	CI
**Antibiotic Escalation yes**	Bone Reconstruction	Yes vs no	**0.008**	2.011	0.67	1.200–3.370
	Radiation	Yes vs no	0.2	1.424	0.35	0.828–2.448
	Clinical SSI	Yes vs no	** < 0.001**	5.469	1.699	3.141–9.520
	Culture positive SSI	Yes vs no	**0.03**	2.9	1.068	1.117–7.583
	LOS ICU	Days	**0.01**	1.121	0.15	1.023–1.228
	HAP	Yes vs no	**0.01**	5.920	1.778	1.562–22.44
**Antibiotic > 10 days yes**	Bone Reconstruction	Yes vs no	**0.06**	2.016	0.7	1.228–3.316

*LOS ICU* Length of stay Intensive care unit, *SSI* Surgical site infection, *HAP* hospital acquired pneumonia

**Table 3:** Multivariate linear regression ANOVAS

Total antibiotic duration		B	***p***-value	CI
	Radiation	1.950	** < 0.001**	0.965–2.935
	Bone Reconstruction	2.506	** < 0.001**	1.569–3.442
	Culture positive SSI	3.536	** < 0.001**	2.234–4.838

*SSI* Surgical site infection

## Discussion

The aim of our study was to identify independent factors that predicted a prolongation of the calculated perioperative antibiotic treatment leading in to therapy and to identify predictors for failure of the calculated antibiotic treatment and subsequent escalation to another preparation.

Among others we were able to demonstrate, that SSIs lead to escalation of antibiotic therapy regardless of bacterial culture positivity. Furthermore, mandibular bone reconstruction could be considered a predictor for antibiotic escalation and prolongation over 10 days postoperatively.

The aim of perioperative antibiotic prophylaxis in free flap reconstruction, is to prevent nosocomial infections like SSI and hospital acquired pneumonia which account for up to 20% of postoperative complications and determine microvascular success [4, 5, 11, 17, 23]. Systematic reviews considering perioperative prophylaxis in general head neck surgery suggest a single dose of intravenous antibiotics during surgery, followed by oral antibiotics is suitable [5, 24, 25]. However, these guidelines do not fully address the complexities of surgeries involving microvascular free flap reconstruction and few prospective studies have shown clear protocols for shortening the duration of antibiotic use in these complex surgeries. Consequently, clinicians tend to postoperatively extend antibiotic prophylaxis up to 10 days or longer to prevent nosocomial infections after free flap surgery indistinctly transitioning into postoperative antibiotic therapy [26].

It has been demonstrated that nosocomial infections, particularly surgical site infections can arise despite or due to inappropriate perioperative antibiotic use after head neck surgery [4]. Ultimately, it remains unclear why some patients experience poor outcomes despite all precautions, resulting e.g. in the failure of reconstruction. Synoptically this might be explained either with incorrect antibiotic selection or through the disruption of microbial niches, allowing virulent bacteria, which were previously suppressed by commensals, to postoperatively proliferate, cause SSI or remote site infections like HAP and leading to disintegration of microvascular reconstructions or deterioration of the general condition, with respiratory failure extending to death [12, 16, 17, 27–30].

With around 25% incidence of cultural positive SSI in our cohort we find the results to be coherent to the literature [4]. Our data indicates that in a total of 25% patient cases, the initially calculated perioperative antibiotic preparation had to be escalated. In 61% of the patients this escalation was necessary due to a clinically suspected SSI with consequent microbiological testing, with around 90% of these tests led to a culture positive SSI with a corresponding antibiogram. Overall, in 27 cases it was not possible to transform the clinical infection into a cultural antibiogram. Early escalation of antibiotics followed by a delayed swab test may result in a “false negative” microbiological culture. This might reduce the bacterial density to a level preventing cultural bacterial growth [31]. According to Rasnake et al., culture-negative SSIs are particularly challenging to treat since treatment options and antibiotic escalation rely on clinical experience rather than on resistance profiling [32]. Therefore, adherence to established infectious disease protocols, including preoperative bacterial screening, is recommended [33]. In reconstructively demanding salvage cases, the authors suggest to supplement preoperative diagnostics with molecular techniques like 16s RNA sequencing [31].

In our multivariate analysis, we found that both suspected surgical site infections, without microbiological confirmation and culture positive infections led to antibiotic escalation. This indicates that clinicians prioritize their clinical examination over microbiological tools, when deciding to escalate antibiotic medication. In our cohort, 43 patients underwent antibiotic escalation for other reasons than clinical surgical site infections. Of these cases, 46% involved resections located in the lower jaw, with 86% being tumor patients and radial forearm and fibula flaps being the most commonly utilized. Additionally, 6 patients from this group developed hospital-acquired pneumonia. This trend may be attributed to SSIs that were not microbiologically confirmed, complications with the anastomosis necessitating revision and subsequent prophylactic enhancement of antibiotic coverage or simply the surgeon’s preference. Hamilton et al. demonstrated that abnormal laboratory results are more likely to contribute to antibiotic escalation than negative cultures are to prompt de-escalation in a hospitalized setting [34]. These finding should encourage improvements in clinicians’ implementation and interpretation of microbiology results.

Statistically antibiotic treatment was mostly escalated to Piperacillin/Tazobactam (53.1%) and in some cases to Ciprofloxacin (18.9%). Literature indicates that SSIs in clean-contaminated surgery frequently involve polymicrobial bacterial spectra [4, 35]. This explains the calculated escalation to a Piperacillin (53%) formulation in our cohort, which typically provides broad-spectrum coverage, especially for Gram-negative bacteria [36, 37]. Ciprofloxacin is frequently used when Pseudomonas is detected, even though the relative prevalence in our study was rather low with 4% [38].

Under multivariate analysis, culture-positive SSIs significantly prolonged the total duration of antibiotic application, with an odds ratio of 3.5 (12.3 vs. 17.3 days) (Table 3). Numerous studies have demonstrated that the use of antibiotics, particularly broad-spectrum antibiotics, is associated with significant adverse effects. Gastrointestinal complications, including diarrhoea and C. difficile infections, as well as Candida overgrowth, particularly in patients with diabetes, are.

well-documented [39]. Additionally, evidence suggests an elevated risk of cardiac mortality in women who consume antibiotics for even less than 15 days during adulthood [40]. We know from literature reviews in other medical fields that it is not uncommon to see delayed de-escalation and hesitant transition to oral therapy resulting in antibiotic mis and overuse [41, 42]. Furthermore, studies have shown that prolonged antibiotic use offers no benefit to tumor patients at all. Instead, those needing adjuvant therapy after ablative tumor surgery benefit from the shortest possible perioperative antibiotic duration, as extended use can reduce response rates to adjuvant therapy and significantly shorten overall survival [13, 14]. The dogma of perioperative antibiotic prophylaxis “one-size-fits-all” should be tailored to the specifics of the surgical procedure, the patient and the unique aspects of the reconstruction, allowing for safe discontinuation of antibiotics after a short period.

Additionally in univariate analysis, haemoglobin levels and postoperative transfusion were found to correlate with antibiotic escalation (Table 1). However, this association was not confirmed in the multivariate model. It is recognized that low haemoglobin levels and subsequent transfusions are risk factors for nosocomial infections [43, 44]. The immune system may be directly compromised by a reduced oxygen-binding capacity otherwise low haemoglobin levels often co-occur with other comorbidities. Direct interactions between erythrocyte concentrates and the host immune system after transfusion is also a subject of ongoing discussion [45–48]. Nevertheless, it is evident that patient blood management, particularly in oncology patients, can enhance patient outcome, shorten overall hospital stay, and simultaneously reduce the risk of nosocomial infections [49].

Examining multivariate factors in our cohort that lead to an escalation or prolongation of calculated perioperative antibiotics, the most notable factor is microvascular bone reconstruction (Tables 2 and 3). Previous studies on this patient population have highlighted that managing patients with mandibulectomy and microvascular bone reconstructions poses significant challenges and is associated with increased all over complication rates [4, 50, 51]. This may be attributed to the prolonged duration of surgery, extensive incisions and the complex three-dimensional nature of the defect. Our data indicate that segmental mandibulectomy (*p* = 0.02) necessitates significantly longer durations of antibiotic therapy compared to partial mandibulectomy (Fig. 2). While the causality between SSIs and the use of small titanium plates remains unclear, a significantly higher complication rate is reported with the use of solitary reconstruction plates following segmental mandibulectomy suggesting that immediate microvascular reconstruction may yield superior outcomes [52]. Regarding preoperative radiotherapy, length of stay in the intensive care unit and nosocomial pneumonia, these factors are well-recognized risk factors for more complicated hospitalizations and impairment of microvascular reconstructions. Preoperative irradiation is a risk factor for worse outcomes following head and neck surgery, including flap loss and nosocomial infections [4]. In addition to impaired wound healing, there is a significantly increased likelihood of surgical site infections, likely attributable to alterations in the oral microbiome induced by radiation [8, 23]. For management targeted interventions, such as elective perioperative tracheotomy to shorten postoperative ventilation and preoperative dental rehabilitation with tooth extraction is controversy discussed [53–55].

In our retrospective analysis of antibiotic application time, the median duration was 10 days, with an average exceeding 12 days (Table 1). These findings require critical discussion, particularly regarding the transition from prophylaxis to therapy. The literature struggles to distinct clearly between prolonged perioperative antibiotic prophylaxis and the initiation of actual postoperative antibiotic treatment. As demonstrated by Mitchell et al. in a large cohort study of 427 patients undergoing microvascular head and neck reconstruction, the duration of postoperative antibiotic administration seems highly variable. In their study, only 23% of patients received antibiotics for 24 h or less, while the majority were treated for > 7days, with some exceeding 20 days [56]. The authors referred to this as prolonged perioperative antibiotic use and reported that patients receiving extended antibiotic prophylaxis had a significantly reduced risk of SSI [56]. However, the point at which prophylactic antibiotic use transitions to therapy remains poorly defined.

Our findings suggest that perioperative antibiotic prophylaxis, followed by postoperative therapy for over 10 days in at least 27% of our cohort, may be unnecessary in the absence of signs for clinical infection since there is no correlation avoiding SSI or HAP. Current systematic studies, such as those by Haidar et al., recommend that adequate perioperative antibiotic prophylaxis following microvascular head and neck reconstruction should not exceed 24 h [24]. However, there is evidence suggesting that patients with altered microbiomes—such as those who have undergone radiation therapy or mandibular reconstruction after osteonecrosis —might benefit from longer prophylaxis but remains vague due to the lack of prospective studies on this topic [4]. This may explain why clinicians in our study, as well as in other referenced studies, hesitated to discontinue perioperative antibiotic administration despite the absence of clinical evidence for surgical site infections.

This “dilemma” of extended postoperative antibiotic administration is likely attributable to the complex nature of the reconstructive cases within our patient population and missing antibiotic stewardship programmes in the past [57]. Studies on antibiotic therapy indicate a consensus towards minimizing the duration of antibiotic therapy, despite the considerable heterogeneity observed in some of the patient groups studied [58]. Prolonged antibiotic use is well-documented to promote the selection of resistant pathogens, inducing highly resistant mutans which can subsequently lead to surgical site infections and necessitate further escalation of antibiotic treatment [59–61]. In irradiated patients literature lacks systematic knowledge of patient’s oral microbiome, missing clear protocols for perioperative or neoadjuvant antibiotic therapy [62]. This underscores a significant gap, as it is well-established that the intraoral microbiome of patients with tumours or osteonecrosis, who constitute the core of the reconstructive patient population, can substantially differ from that of healthy individuals [7, 10, 63]. Such patients may harbour bacteria that, due to localized selection pressures, prolonged antibiotic use or radiotherapy do not conform to standard perioperative therapy protocols, leading to selective enrichment of these resistant strains after perioperative antibiotic prophylaxis. This necessitates further molecular biological studies to enable a systematic analysis of this reconstructive cohort and to derive evidence-based recommendations for pre-emptive antibiotic treatment.

However, the implementation of antibiotic stewardship guidelines in surgical disciplines remains yet limited [64]. To address this issue and reduce antibiotic duration, several key measures are necessary, particularly in complex reconstructive cases with a history of radiation or bone reconstruction. These include establishing local treatment protocols, monitoring antibiotic duration, optimizing dosage, and ensuring timely transitions to oral therapy. Additionally, collaboration with infectious disease specialists and clinical microbiologists could enable preoperative microbiome analysis to identify potential resistant pathogens, thereby allowing for more targeted antibiotic therapy adjustments[65, 66].

In summary, the authors conclude that the study’s results must have direct clinical implications. Firstly, a structured Antimicrobial Stewardship program needs to be established, which defines clear and reproducible protocols for perioperative antibiotic prophylaxis to prevent the increase in multidrug-resistant organisms and antibiotic-associated side effects. Patients with specific risk factors, such as preoperative radiation therapy or colonization with resistant organisms, should be identified preoperatively to determine those who may benefit from extended postoperative antibiotic coverage. Additionally, patients who are more likely to experience harm from prolonged therapy should be identified, as a short perioperative prophylaxis would suffice for them. A stricter Surgical Site Infection surveillance system will help identify microbial spectra, ensuring that even after multiple reconstructions, patients receive appropriately tailored perioperative antibiotic coverage.

## Limitations

This study has several limitations. Due to its retrospective nature, the data and statistical analysis are dependent on the accuracy and completeness of the patient data documentation. As a single-center study, only correlations rather than causalities, can be transferred to the general population. The diagnosis of surgical site infections is based on the clinical patient presentation which can vary significantly between individuals. Furthermore, microbiological diagnostics are subject to preclinical sampling errors, potentially leading to an underestimation of the incidence of culture-positive SSIs. Antimicrobial Stewardship guidelines have been recently implemented in our clinical practice, which must be considered interpreting data.

## Conclusion

Surgical site infections are a significant driver of escalation in perioperative antibiotic use. Clinical suspicion alone is often sufficient to warrant escalation, even in the absence of positive microbiological findings. Microvascular bone reconstruction predicts for both escalation and the continuation of perioperative antibiotics for more than 10 days. Additionally, preoperative irradiation, the presence of hospital-acquired pneumonia and extended stay on intensive care unit are associated with increased escalation probability resulting in significantly prolonged antibiosis administration during hospitalization. The implementation of a structured Antimicrobial Stewardship is mandatory to reduce excessive postoperative antibiotic coverage and prevent colonization with multidrug-resistant organisms leading to antibiotic escalation.

## Data Availability

No datasets were generated or analysed during the current study.

## Abbreviations

ICU: Intensive care unit
NW: Normal ward
LOS: Length of stay
SSI: Surgical site infection
HAP: Hospital acquired pneumonia
ASA: Physical status classification system of American Society of Anaesthesiologists
HB: Haemoglobin
RFF: Radial forearm flap
FFF: Free fibula flap
ALT: Anterior lateral thigh flap
LAT: Latissimus flap
UA: Upper arm flap
DCIA: Deep circumflex iliac artery bone flap
